# Disparate behavioral types in wild and reared juveniles of gilthead seabream

**DOI:** 10.1038/s41598-023-37554-7

**Published:** 2023-07-11

**Authors:** Javier Sanllehi, Marco Signaroli, Aina Pons, Martina Martorell-Barceló, Júlia Mulet, Arancha Lana, Margarida Barcelo-Serra, Eneko Aspillaga, Amalia Grau, Ignacio A. Catalán, Tomeu Viver, Josep Alós

**Affiliations:** 1 Instituto Mediterráneo de Estudios Avanzados, IMEDEA (CSIC-UIB), C/Miquel Marquès 21, 07190 Esporles, Illes Balears Spain; 2 Laboratori d’Investigacions Marines i Aqüicultura d’Andratx, LIMIA (IRFAP), Avinguda de Gabriel Roca i Garcías, 69, 07157 Andratx, Illes Balears Spain

**Keywords:** Behavioural ecology, Zoology

## Abstract

Fish differ consistently in behavior within the same species and population, reflecting distinct behavioral types (BTs). Comparing the behavior of wild and reared individuals provides an excellent opportunity to delve into the ecological and evolutionary consequences of BTs. In this work, we evaluated the behavioral variation of wild and reared juvenile gilthead seabreams, *Sparus aurata*, a highly relevant species for aquaculture and fisheries. We quantified behavioral variation along the five major axes of fish behavioral traits (exploration-avoidance, aggressiveness, sociability, shyness-boldness, and activity) using standardized behavioral tests and a deep learning tracking algorithm for behavioral annotation. Results revealed significant repeatability in all five behavior traits, suggesting high consistency of individual behavioral variation across the different axes in this species. We found reared fish to be more aggressive, social and active compared to their wild conspecifics. Reared individuals also presented less variance in their aggressiveness, lacking very aggressive and very tame individuals. Phenotypic correlation decomposition between behavioral types revealed two different behavioral syndromes: exploration-sociability and exploration-activity. Our work establishes the first baseline of repeatability scores in wild and reared gilthead seabreams, providing novel insight into the behavior of this important commercial species with implications for fisheries and aquaculture.

## Introduction

Animals differ consistently in several aspects of their behavior between members of the same species, cohort, and sex^1–3^. When these individual differences are consistent across time and ecological contexts, individuals reflect distinct “personalities” or behavioral types (BTs) within populations^4–6^. Animal BTs are widespread and have been documented in multiple taxa, including fishes^7^. The degree of consistency in the among-individual behavioral differences is usually quantified with the repeatability (*R*) score, which describes the proportion of phenotypic variance due to among-individual differences^8^. A meta-analysis across taxa (98 species) showed an average *R*-score in different behaviors of 0.37, with the vast majority of scores lying between 0.1 and 0.8^9^.

In animals, the intraspecific behavioral variation is usually described along five major axes of personality, derived from the human Five-Factor Model^10^: exploration-avoidance, aggressiveness, sociability, shyness-boldness, and activity^11^. BTs usually covary forming behavioral syndromes, defined as suites of correlated behaviors expressed within a given behavioral context or across different contexts^1^. Despite the existence of behavioral syndromes, individuals do not exhibit full consistency in their behavior. These syndromes display a certain degree of flexibility, which can prove advantageous in response to changing conditions^12^. For example, Sol et al.^13^ observed that successful invasive bird species exhibited a higher frequency of foraging innovations. Also, populations of bold and aggressive three-spined sticklebacks (*Gasterosteus aculeatus*) were found to be favored in resource-competitive environments, but were at a disadvantage in risky situations due to increased predation risk^12^.

Animal BTs affect an individual at nearly every stage of development but are particularly relevant in juvenile stages as individuals are exposed to many behavioral trade-offs which, in general, tend to maximize growth, survival, and dispersal^14^. For example, pre-settlement schooling behavior in reef fish can influence their dispersal and the distribution of their adult populations^15^. Although some aspects of juvenile behavior are precursors of adult behavior, they often change during the development in response to changing selective pressures^14^. For instance, Carere et al.^16^ demonstrated that manipulating food quantity given to great tit nestlings (*Parus major*) resulted in divergent effects on adult exploratory and aggressive behavior for individuals of distinct lines. These examples show that BTs are involved in many important ecological and evolutionary processes in animal populations^17^, including domestication.

Domestication is the process of adapting wild plants and animals for human use, selectively breeding animals over generations to select for traits that are desirable to humans. As a result, domesticated animals have undergone significant changes in their biology, including behavior^18^. The process of animal domestication varies depending on the species and the diverse objectives for which an animal is reared. For example, the domestication of dogs resulted in the fixation of behavioral traits, such as tameness and social tolerance, which have facilitated their coexistence and cooperation with humans^19^. In fish, domestication selects traits that are beneficial for aquaculture and fish farming. The definition of a domesticated fish is not consistent among authors, but typically refers to a fish that has undergone selective breeding and exhibits a high degree of human control over its life cycle^20^. Fish domestication tends to make organisms more suitable for aquaculture environments, typically small, confined and simple, with abundant food and very dense populations constantly protected from natural predators^21^. Domestication in fish is usually associated with a general acceleration of individual development due, on one side, to the artificial selection (and genetic manipulation) performed by the breeders and, on the other, to the relaxation of natural selection (optimal physical environment, abundance of food and no predator pressure)^22^. The artificial environment also modifies behavioral traits: foraging, anti-predator and reproductive behavior tend to be reduced in complexity and effectiveness, territorial behavior is suppressed or heavily altered, and aggressiveness is usually increased, especially where food is distributed in a localized and predictable manner^21^.

In this study, we examine the degree of behavioral consistency in wild and reared individuals of juvenile gilthead seabreams, *Sparus aurata*. The gilthead seabream is a relevant inshore fish for coastal fisheries of the Mediterranean Sea and the North-east Atlantic Ocean, with annual captures from fishing vessels rounding the 8200 tons in 2019^23^. It is the most economically valuable species among the Sparidae family, and it has become a major resource for the Mediterranean aquaculture sector, with a production estimated at 258,754 t in 2019 (6588 t in Spain in 2020) and with an annual growth of 13.2%^23^. The gilthead seabream underwent initial trials on selective breeding in the mid-1990s, with commercial breeding programs being initiated in the early 2000s. Today, it is considered one of the most domesticated fish species in Europe, with its life cycle being fully controlled in captivity^20, 24^. The gilthead seabream is one of the few marine fish species for which individual behavioral consistency has been demonstrated^25^. However, only a few studies have investigated the BTs of this species and focused only on domesticated strains^25–27^. For example, Castanheira et al.^25^ observed that in juvenile farmed gilthead seabreams the risk-taking and escape behaviors (exploration-avoidance axis) were significantly consistent over time and contexts. Comparing the behavior of wild and reared individuals provides an excellent opportunity to delve into the ecological and evolutionary consequences of BTs by shedding light on the behavioral consequences of domestication.

In this context, the objective of this study is to quantify, through standardized behavioral tests in experimental arenas, the five axes of fish BTs in two samples of juvenile gilthead seabreams (wild and reared), to determine the behavioral consistency at the individual level (repeteability) and the effects of domestication on the behavior of this species. Establishing a behavioral baseline for the gilthead seabream can help us to understand the effect of domestication and the evolutionary and ecological consequences of BTs in this species. A good understanding of the fundamental behavior of wild and reared individuals can bring major improvements to restoration and reintroduction strategies in a fisheries management context^11, 28^ and can give us useful behavioral indicators for fish welfare in fish farms^27, 29^.

## Materials and methods

**Figure 1:**
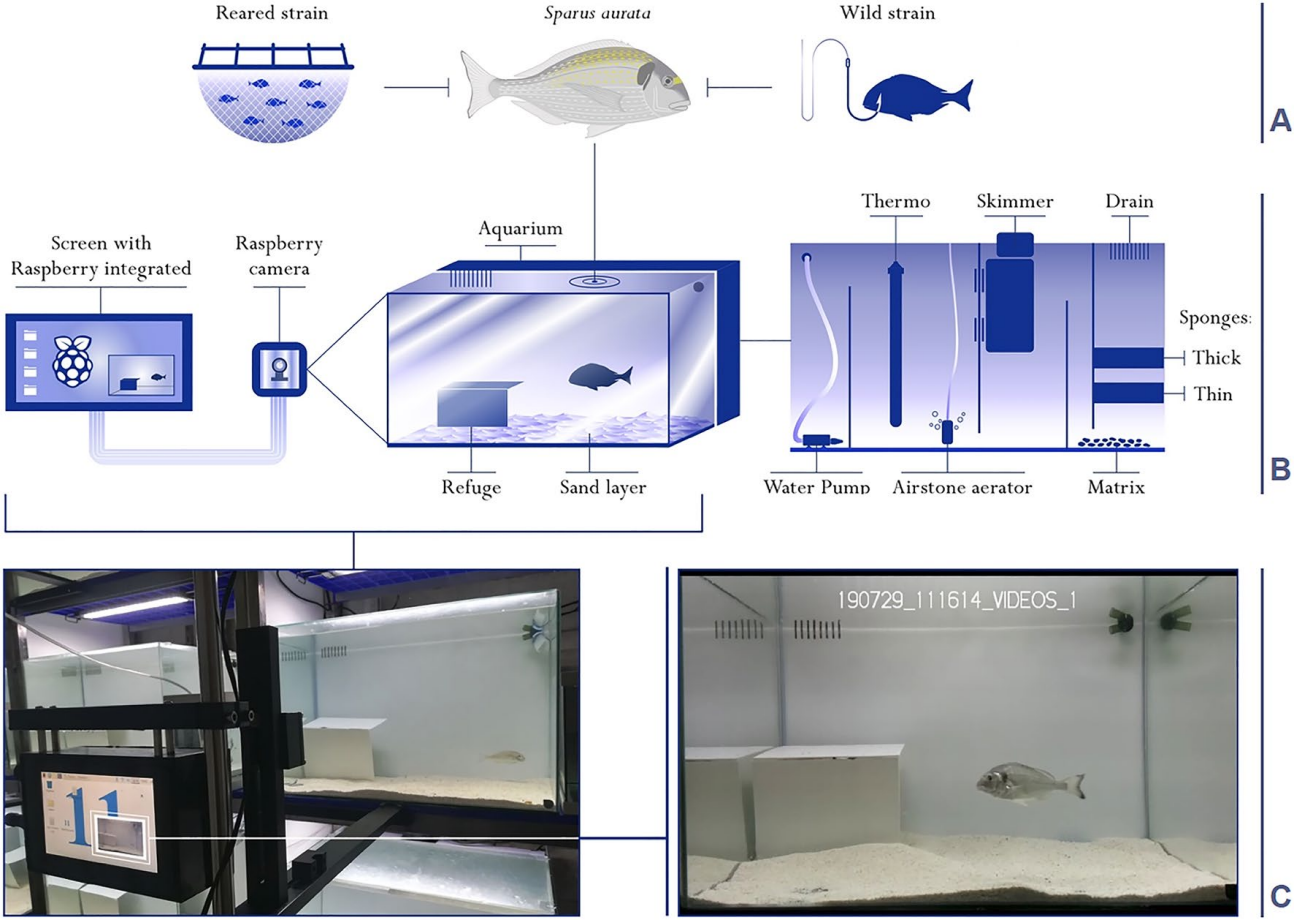
Representative diagram of the experimental behavioral arenas and monitoring equipment. (**a**) Experimental individuals (*Sparus aurata*) were from wild and reared samples. (**b**) Behavioral arenas were composed by a seawater aquarium with their own sump and filter system. The arena presented a sand layer bed and a refuge. (**c**) An integrated recording and tracking systems formed by a Raspberry Pi computer and camera was mounted in front of the arena to measure the 2-dimensional movement of the individuals in the open area.

To quantify within and between-individual variation along the five axes of behavior in the two different samples (wild and reared, Fig. 1a) of gilthead seabreams, standardized behavioral tests were performed in isolation and under controlled conditions at the Marine Research and Aquaculture Laboratory (LIMIA-IRFAP) located in Port d’Andratx (Mallorca, Spain). An experimental room was prepared to host 12 behavioral arenas (aquariums, Fig. 1b), each one with an isolated gilthead seabream. Each behavioral arena was composed of a main aquarium (60 × 50 × 40 cm, 120 L) enriched with a shelter and a sandy bottom (1 cm deep, with grains sized 0.5–1.2 mm), filled with sterilized seawater maintained at 21 °C and constantly cleaned by a filtering system (sink) composed by physical and biological filters, including a Skimmer, re-circulation and an aerator (Fig. 1b). Continuous recording was provided by a camera attached to each arena controlled by a Raspberry Pi 3 system (Fig. 1c). The experimental individuals remained in the arenas for a total of seven days. The fish remained undisturbed for the first three days to acclimate to the new conditions. After this period, every day for the subsequent four days, we performed a standardized behavioral test (see below) for each of the five behavioral axes to obtain repeated measures for each individual. The tests started with wild individuals on March 11th, 2019 and ended on April 23rd, 2019. Reared individuals were tested starting on July 19th, 2019 and ending on August 22nd, 2019. All animal care and laboratory procedures were approved by the Ethical Committee for Animal Experimentation of the University of the Balearic Islands (ref. CEEA 98/07/18) and authorized by the Animal Research Ethical Committee of the Conselleria d’Agricultura, Pesca i Alimentació and the Direcció General de Pesca i Medi Marí of the Government of the Balearic Islands. All the methods were performed in accordance with the European Directive (2010/63/UE) and Spanish Royal Decree (RD53/2013) to ensure good practices for animal care, health, and welfare. The study is reported in accordance with ARRIVE guidelines.

### Origin of the experimental individuals

Two samples of animals from different origins were used for the experiments. The wild sample was composed of 31 individuals captured in the waters of Mallorca with the same standardized gear (one single hook attached to a line with a fishing float) and using the same bait (pieces of bread). In March 2019, twelve individuals per week were captured, transported to the LIMIA (IRFAP) using an oxygenate tank (50 L), and directly isolated in the experimental arenas. The reared sample was provided by the Institute of Agrifood Research and Technology (IRTA, Spain) in July 2019. Two hundred individuals from the same breeding program were transported to the LIMIA (IRFAP) and placed in two 1000 L aquaculture tanks where fish remained undisturbed and fed with conventional pellet food (D-2 OptiBream AE 1P and D-4 OptiBream AE 2P). Then, every 7 days, 12 experimental individuals were randomly selected and introduced into their experimental arenas for the behavioral tests. During the experimental period, wild seabreams were fed three shrimps a day per individual (similar to their natural diet), while the reared ones were fed with 1 g of pellet a day per individual according to the standardized ad libitum protocols for the species. In total, 68 experimental individuals were tested (n = 31 wild and n = 37 reared) with average body lengths of 12.15 ± 1.01 cm and an average weight of 26.69 ± 6.58 g.

### Continuous recording system for behavioral annotation

All the behavioral tests (see below for a description) were recorded and analyzed using the trained deep learning algorithm described in Signaroli et al.^30^ to automatically extract behavioral information. The recording system was composed of a Raspberry Pi 3 (model B+) and a camera (Raspberry Pi V2 camera module of 8 MP) attached in front of each arena (Fig. 1c). The Raspberry Pi is a low-cost single-board computer of small dimensions, with a Linux operating system (Raspberry OS) installed on a Micro SD card. The Raspberry was housed in a plastic case to prevent water damage and equipped with a 7-inch touchscreen display. We programmed our system to continuously record 1080p videos at 30 fps and save them on an external 1 TB hard disk. To automatically track the movements of the fish in the recordings (2-dimensional), we trained a deep learning neural network to detect and locate the gilthead seabream in images (video frames). We used a neural network called Faster Region-based Convolutional Neural Network (Faster R-CNN) that takes as input video frames (in our case, one per second) and predicts the position of the target object (the fish) with an associated probability score^31^. During the training phase, we presented to the neural network 14,000 manually labeled images (the labels are .xml files specifying the position of the fish in every image) taken from the recordings, from which it learned to locate the target automatically. After the training, we validated the functioning of the trained Faster R-CN N, analyzing the same videos both manually (14 videos, a total of more than 50,000 frames) and with the algorithm, and comparing the two results. The neural network reached an accuracy of 92.8% in predicting the presence of a fish in a frame and 98.9% in positioning it, proving to be a useful tool to obtain data on individual fish behavior.

### Standardized behavioral tests

After the acclimation period in the experimental behavioral arena, fish were familiar with the new environment and showed normal swimming behavior. The objective of the standardized behavioral tests was to obtain four repeated measures per individual (one per day) for the five behavioral axes: exploration-avoidance, aggressiveness, sociability, shyness-boldness, and activity (Table 1). Each test lasted one hour, except the activity test that lasted 2 h to collect enough locomotory data since no human intervention was needed during this test. Between the tests, half an hour was left for the individual to rest, remaining undisturbed. Activity was measured using morning videos before the tests started. The shyness-boldness test was always the last test carried out each day as it required feeding the fish (see below). The order of the other three tests was randomized to avoid within-day temporal influence on the assessment of behavior. All the spatial metrics used in the statistical analysis were measured in pixels. However, to present the results, we estimated their equivalence in centimeters, using the mean pixel value of the 60 cm between the back and the front side of the arena (1 cm = 16.7 px). The five standardized behavioral tests were carried out as follows (Table 1):

**Table 1:** Design, representation, time (duration of the test in hours) and generated behavioral metrics of the tests on the five axes of behavior variation.

Axis	Test	Design	Representation	Time	Metrics
Exploration-avoidance	Novel object test	Introduce a novel object into the experimental arena		1 h	Number of attempts (per 1-h)
					Time outside the refuge (s)
					Minimum distance to object (px)
					Time interacting with the novel object (s)
Aggressiveness	Mirror image stimulus test	Place a mirror in contact to the experimental arena		1 h	Number of attempts (per 1-h)
Sociability	Separation test	Place a secondary aquarium with a conspecific individual next to the experimental arena		1 h	Time outside the refuge (s)
					Minimum distance to the other individual (px)
					Total time interacting with the other individual (s)
Shyness-boldness	Predador stimulus test	Scare the individual with a hostile object and then fed		1 h	Head latency (s)
					Body latency (s)
					First bite latency (s)
Activity	Open-field test	Quantify the movement with automatic tracking		2 h	Travelled distance (px)
					Time outside the refuge (radians)
					Total time travelled (s)
					Average turning angle (radians)
					Area used (px)
					Core area used (px^2^)

#### Exploration-avoidance measurement using the novel-object test

Several studies have assessed the individual consistency in exploratory behavior of fish using the novel-object test^32, 33^. In our tests, a small colored toy animal figurine was introduced into the arena to test the exploration-avoidance of the individual exposed to the presence of the new item (Table 1). For all tests, the novel object was placed at the center of the arena next to the front glass. We quantified the exploration-avoidance behavior through two groups of metrics: first, we manually counted (visualizing the videos) the number of ”approaches”, defined as fast approaches with physical contact to the novel object; and second, we extracted several metrics from the data generated by the automatic tracking: (i) time outside the refuge during the test, (ii) minimum distance to the object, measured as the minimum distance between all positions of the fish and the centroid of the novel object in pixels, and (iii) total time interacting with the novel object, measured as the time during which the fish was inside a radius of 100 pixels (~ 6 cm) from the centroid of the novel object. In the case of the minimum distance, whenever the fish was not detected (i.e. remained in the refuge), a distance of 250 pixels (~ 15 cm, that is, the average distance between the novel object and the refuge) was assigned to it. Since the individuals were tested for four consecutive days, we used a different toy figurine every day to ensure there was no previous information on the novel object that could affect behavior.

#### Aggressiveness measurement using the mirror test

Aggressiveness in fish is mainly measured by mirror image stimulus via aggressive displays or direct contact^5, 34–36^. In our study, a mirror was placed in contact with one side of the behavioral arena, simulating the presence of a conspecific at close range (Table 1). We measured the number of aggressive and hostile "approaches" the fish gave to the mirror per hour by manually visualizing the videos^36, 37^**.** We defined a hostile “approach” as a fast movement with physical contact to the mirror, including instances of attempted biting and the extension of the dorsal fin^36^.

#### Sociability scoring using a social cue

Sociability is mainly quantified by separation tests, measuring the individual reaction to separation from a group or its latency in joining a group from isolation^5, 38, 39^. We measured sociability by exposing the target individual to a similar size individual, randomly chosen from the two aquaculture tanks, and placed inside a secondary aquarium next to the main experimental arena (Table 1). The glass separation allowed visual interaction, excluding physical or olfactory, between the individuals. Behavioral scoring for sociability was entirely based on the tracking data, from which we extracted three metrics: (i) time outside the refuge during the test, (ii) minimum distance to the other individual, measured as the minimum distance between all positions of the fish and the centroid of the other individual in pixels, and (iii) total time interacting with the conspecific, measured as the time spent inside a 100 pixels (~ 6 cm) radius from the centroid of the other individual. In the case of the minimum distance, whenever the individual was not detected (i.e. remained in the refuge), a distance of 350 pixels (~ 20.1 cm) was assigned, according to the average distance between the shelter and the conspecific.

#### Shyness-boldness using the predator stimulus test

Previous research has considered several methods to measure boldness in fish, such as the latency to explore a novel environment or object under a risky situation^40^. However, considering the ecological implications of boldness, the response to a predator stimulus has been found as the most useful method^12, 41^. To recreate a risky situation, we simulated a predator attack by disturbing the individual with an object (an aquarium clamp, always introduced from the same angle) for five seconds. After the attack, we delivered the daily food item and measured the behavioral response (Table 1). Behavioral scoring of the shyness-boldness trait was based on three different metrics: (i) the head latency, or the time between the simulated attack and the first time the head of the fish was detected out of the refuge, (ii) the body latency, or the time between the simulated attack and the moment the fish left the refuge and (iii) the first bite latency, or the time between the simulated attack and the first time the fish bit the food^42^. All these metrics were manually collected visualizing the videos.

#### Activity measured through the open-field test

The activity can be quantified using the open-field tests by measuring the distance covered by the individual during a given time^5, 43^. To measure the basal activity, two hours of video were chosen from the morning recordings before the tests started. We generated three different metrics from the trajectories obtained with the neural network: (i) time outside the refuge during the 2 h, (ii) total distance traveled (in pixels), adding the Euclidean distances between two consecutive positions of the fish, (iii) average turning angle (preferred direction, in radians) and concentration (in radians), generated by fitting all turning angles to Von Mises probability distribution (a reciprocal measure of dispersion and tortuosity, in radians), (iv) area used, the area of pixels that has a 95% probability of containing the fish, and (v) core area, the area of square pixels that has a 50% probability of containing the fish. All metrics were calculated for individuals that generated at least 20 positions (i.e., individuals that were detected outside the refuge for at least 20 s) to ensure a representative sample size.

## Data-analysis

### Contextual variance of behavior

Several variables were considered in the data analysis of fish BTs. Fish total length and weight were highly correlated (GLM: T-value = 34.11, *p*-value < 0.001). Thus, length was only incorporated as a fixed factor in the statistical analysis model to avoid co-linearity issues. However, its effects were interpreted in conjunction with the effect of weight. Four experimental trials (one trial consisting of five tests) were conducted on four consecutive days per individual; therefore, to control for a possible learning bias, the trial number was included as a fixed factor. The statistical model also incorporated fish sample origin (wild vs. reared). We fitted linear mixed models (LMM) using the R-package MCMCglmm^44^ following the general procedures proposed by Dingemanse and Dochtermann^45^ and Harrison et al.^46^ to properly decompose the raw phenotypic variance for each single behavioral trait metric. Principal component analyses (PCAs) were run to explore covariability among the different metrics generated for exploration, activity, sociability, and boldness (aggressiveness was estimated using only one metric), and to summarize correlated behavioral metrics that collectively explained the largest proportion of the observed variation reducing the number of variables to one univariate LMM per trait. With this procedure, the values of the first principal component (PC1) were used as scores describing the behavioral types of each individual in each trial. Specifically, four PCAs were conducted separately for exploration, sociability, boldness, and activity. We fitted a total of five different LMMs, one for each behavioral trait, to explain the contextual variance, including the fixed factors mentioned above and the identifier of the fish (id) as a random effect (all continuous variables were mean-centered). All models fitted here considered a Gaussian response by exploring the distribution of the residuals, and only exploration and sociability scores were log-transformed to reach normality of residuals. The parameters of the LMMs and the *p*-value were estimated using a Bayesian approach (Monte Carlo Markov Chains, MCMC) with the default setting on iterations (13,000 iterations), burn-in (3000 first set of iterations discarded) and thinning (10 thinning interval to avoid autocorrelation). In all cases, convergence of the chains was attained and checked by plotting the MCMCglmm objects generated^44^. The Bayesian Credibility Intervals (BCI, 2.5% and 97.5%) were estimated for all parameters. The full models (with all fixed effects) were reduced using a step-by-step backward reduction until the higher explanatory power was attained (the lowest Deviance Information Criterion, DIC).

### Adjusted repeatability of behaviors

The repeatability (*R*) score assesses the degree of consistency of behaviors shown by individuals over time^9^. The R-score represents the phenotypic variation attributable to individual heterogeneity and is often used to characterize animal behavioral types^45, 47^; it can be estimated as*:*

1 $$R=\frac{{Vind}_{0}}{({Vind}_{0}+{Ve}_{0})}$$

where ${Vind}_{0}$ represents the between-individual variance (across random intercepts of individuals) and ${Ve}_{0}$ represents the within-individual variance for a given behavioral trait. We used the LMM described in the previous section to decompose the raw phenotypic variance of each single behavioral metric into between- and within-individual variances and computed adjusted-*R* scores for each trait (adjusted repeatability after controlling the confounding fixed effects) following Eq. 1. We included the fixed effects mentioned before and calculated the adjusted-*R*. To examine the consistency of behaviors shown by individuals over time and detect the presence of BTs, we computed the BCI for all adjusted-*R* and used the likelihood ratio tests (LRTs) to calculate the significance of this score. DIC reduction differences between the unconstrained LMM and the constrained LMM larger than 2 were considered significant adjusted-*R*, according to Nakagawa and Schielzeth^47^ and Dingemanse and Dochtermann^45^. Adjusted-*R* scores were interpreted biologically following the previous *R* meta-analysis in Bell et al.^9^. When there were notable dissimilarities in the distribution of scores between the two samples (wild and reared), an F-test was computed to determine any significant variations in their variances.

### Between- and within-individual correlations among behaviors

We used bivariate (paired traits) LMM to decompose phenotypic correlations between paired behavioral traits (*r*_*p*_) into between- (*r*_*ind*_) and within-individual or residual (*r*_*e*_) correlations according to the procedures described in Dingemanse and Dochtermann^45^. This phenotypic correlation decomposition aimed to detect the existence of behavioral syndromes (i.e. correlates suites of behavioral traits^1^). We used the MCMCglmm R-package to fit bivariate (paired traits) LMMs (5 different models) with the recommended flat/uninformative prior structure suggested by Dingemanse and Dochtermann^45^. All significant effects found in the previous section were included in the models. To ensure convergence of the models, we considered 100,000 iterations, thinned every 100 iterations to avoid auto-correlation and discarding the first 10,000 (burn-in). The reduction in the DIC (ΔDIC), provided by the paired LMM where between-individual and residual covariance were constrained to 0, was used to detect significant correlation coefficients (DIC reductions greater than 2 were considered significant^46, 48^).

## Results

A total of four separate PCAs were run on behavioral metrics obtained from the exploration, sociability, boldness, and activity tests, reducing the data into two components for each behavioral trait and then using the first one (PC1) for further analysis. For exploratory behavior, the four behavioral metrics measured were positively correlated and collapsed into a principal component (PC1) used as an “exploration score”, explaining 44.5% of the total variance observed. Therefore, more positive scores described more exploratory individuals that exhibited a higher number of approaches and more time interacting with the novel object, while more negative scores were indicative of avoidance with a larger minimum distance to the novel object. In sociability, the three behavioral measures were correlated and reduced to a “sociability score” explaining 62.4% of the total variance. Positive scores described social individuals with longer times interacting with a conspecific, and negative scores described more asocial traits with a larger minimum distance. For boldness, the three metrics were positively correlated, resulting in a “boldness score” explaining 86.3% of the total variance, where a more positive score was indicative of bold individuals and, on the contrary, a more negative score defined shy individuals (higher latencies). A total of six metrics of activity were represented by the first component (PC1), referred to as “activity score”, explaining 89.0% of the total variance. High score values described more active individuals. We used these newly generated behavioral scores to further analyze repeatability and behavioral syndromes. Regarding aggressiveness, it was not necessary to conduct a PCA since it was quantified using only one metric (number of approaches). Therefore, more aggressive individuals presented higher values on this variable.

### Repeatability and analysis of contextual variance

**Figure 2:**
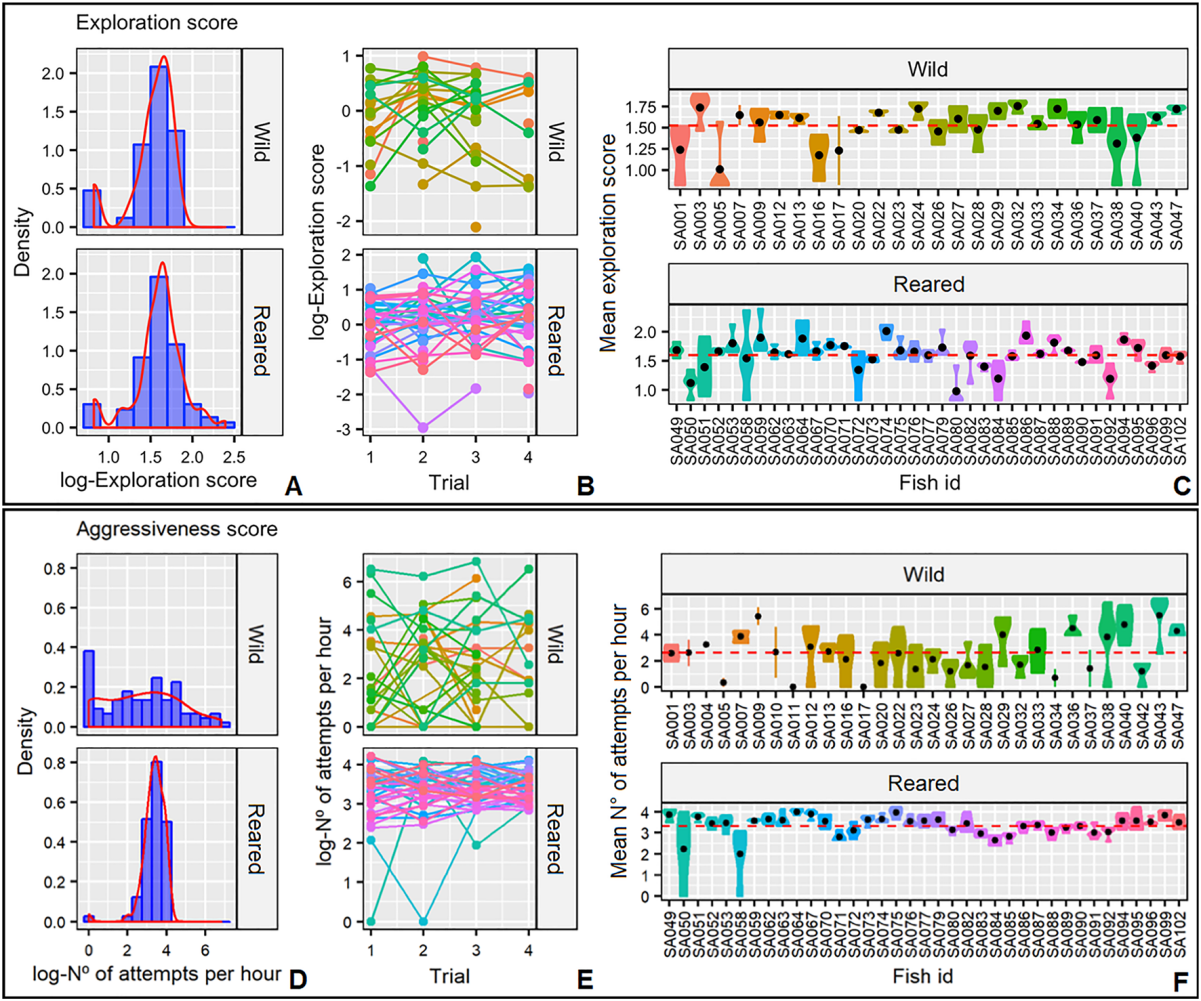
(**a**) Density plots of the exploration score, (**b**) individual’s exploration score per trial and (**c**) individual’s mean exploration score (black dot) for the two samples of gilthead seabream (*Sparus aurata*). Red dashed line represents the respective sample average; (**d**) Density plots of the number of attempts, (**e**) individual’s number of attempts per trial and (**f**) individual’s mean number of attempts (black dot) for the two samples of gilthead seabream. Red dashed line represents the respective sample average.

Adjusted-*R* score for exploratory behavior in wild and reared samples was significant and averaged 0.46 with a BIC ranging from 0.37 to 0.50 (Table 2), denoting a repeatable exploratory behavior in juvenile gilthead seabreams that is consistent between individuals and across time (Fig. 2a–c). Individuals varied greatly in exploration. The most exploratory individual (SA074; Fig. 2c) interacted 45 times with the novel object and approached it with a minimum distance of 33 pixels (1.98 cm). On the contrary, the less exploratory (SA080; Fig. 2c) showed no interactions remaining in the refuge for the entire duration of the four trials (250 pixels). The analysis of the potential covariates affecting exploration suggested that neither individual length nor sample affected this behavior [LMM; *p* = 0.764 (length); *p* = 0.252 (sample); Table 2]. The LMM showed significant differences during the experimental trials (*p* < 0.05; Table 2), with a relative increase in exploratory behavior with the progress of the trials in both samples.

**Table 2:** Covariates (posterior mean shown) with their Bayesian credibility intervals (lower l- and upper u-BCI) and *p*-values (pMCMC) of the five linear mixed models (LMMs) fitted for the five behaviors studied: exploration-avoidance, aggressiveness, sociability, shyness-boldness, and activity.

	Post. mean	I-BCI	u-BCI	pMCMC
Exploration				
Intercept	1.53	1.44	1.63	< 0.001***
Trial	0.03	0.01	0.06	0.012*
Length	− 0.01	− 0.07	0.05	0.764
Sample-reared	0.07	− 0.06	0.18	0.252
* V*_*ind*_	0.04	0.02	0.06	
* V*_*e*_	0.04	0.06	0.06	
Adjusted-R	0.46	0.37	0.50	
DIC = − 11.54 (DIC_C_ = 79.08)				
Aggressiveness				
Intercept	2.63	2.25	3.06	< 0.001***
Trial	0.08	− 0.04	0.21	0.212
Length	0.10	− 0.15	0.37	0.474
Sample-reared	0.70	0.21	1.27	0.004**
* V*_*ind*_	0.66	0.35	1.16	
* V*_*e*_	0.95	0.80	1.21	
Adjusted-R	0.41	0.31	0.49	
DIC = 718.20 (DIC_C_ = 789.90)				
Sociability				
Intercept	− 0.61	− 1.03	− 0.14	0.014*
Trial	0.02	− 0.09	0.14	0.728
Length	− 0.08	− 0.35	0.19	0.522
Sample-reared	0.95	0.36	1.51	< 0.001***
* V*_*ind*_	0.81	0.60	1.41	
* V*_*e*_	0.75	0.60	0.93	
Adjusted-R	0.53	0.49	0.60	
DIC = 636.63 (DIC_C_ = 767.40)				
Boldness				
Intercept	− 2076.20	− 2716.76	− 1505.83	< 0.001***
Trial	− 35.21	− 245.41	151.16	0.698
Length	− 6.23	− 323.26	323.74	0.958
Sample-reared	334.40	− 479.15	1008.68	0.374
*V*_*ind*_	560,745.50	224,199.40	1,108,809.80	
* V*_*e*_	1,697,069.00	1,286,991.00	2,110,943.00	
Adjusted-R	0.25	0.15	0.34	
DIC = 3415.58 (DIC_C_ = 3446.22)				
Activity				
Intercept	2.05	1.86	2.21	< 0.001***
Trial	− 0.06	− 0.12	− 0.01	0.014*
Length	0.05	− 0.08	0.16	0.434
Sample-reared	0.32	0.10	0.56	0.004**
* V*_*ind*_	0.17	0.11	0.25	
* V*_*e*_	0.15	0.12	0.18	
Adjusted-R	0.54	0.47	0.58	
DIC = 266.84 (DIC_C_ = 397.22)				

The table shows the LMM parameters after the reduction according to the maximum explanatory power using the deviance information criterion (DIC). Experimental trial and sample (reared or wild) are categorical variables, while individual length is treated as a continuous variable. The between (*V*_*ind*_) and within individual (*V*_*e*_) variances and the adjusted repeatability (adjusted*-R)* for each trait are also shown. The DIC of the reduced LMM and the DIC of the constrained LMM (DICc) are shown for all behavioral traits. The asterisks on the last column indicate the significance of *p*-values.

Adjusted-*R* estimate for aggressive traits in wild and reared samples was significant and averaged 0.41 with a BIC ranging from 0.31 to 0.49 (Table 2), describing a repeatable aggressive behavior in juvenile gilthead seabreams that is consistent between individuals and across time (Fig. 2d–f). The behavioral variation was reflected by contrasting extreme individuals, where the most aggressive individual (SA043; Fig. 2f) displayed a total of 909 approaches to the mirror in only one trial and, on the opposite side, the less aggressive individual (SA017; Fig. 2f) did not interact with the mirror in any of the trials. The analysis of the potential covariates affecting aggressiveness suggested that neither experimental trial nor individual length affected this behavior [LMM; *p* = 0.212 (trial); *p* = 0.474 (length); Table 2]. The LMM showed significant differences in aggressive interactions of gilthead seabreams between fish samples (*p* < 0.01; Table 2), where the reared sample showed slightly higher levels of aggressiveness. Nevertheless, the disparity in the among-individual variance between the samples limits the robustness of the LMM and the interpretation of the results (Fig. 2d). Wild fish showed higher among-individuals variation in aggressiveness than reared fish, resulting in highly aggressive and very tame individuals. In contrast, reared fish showed less variation, being most individuals similar in their aggressiveness score (Fig. 2d). The results of the F-test indicated a significant difference in the variance of the two samples, with a calculated value of 113.43 (*p* < 0.001).

**Figure 3:**
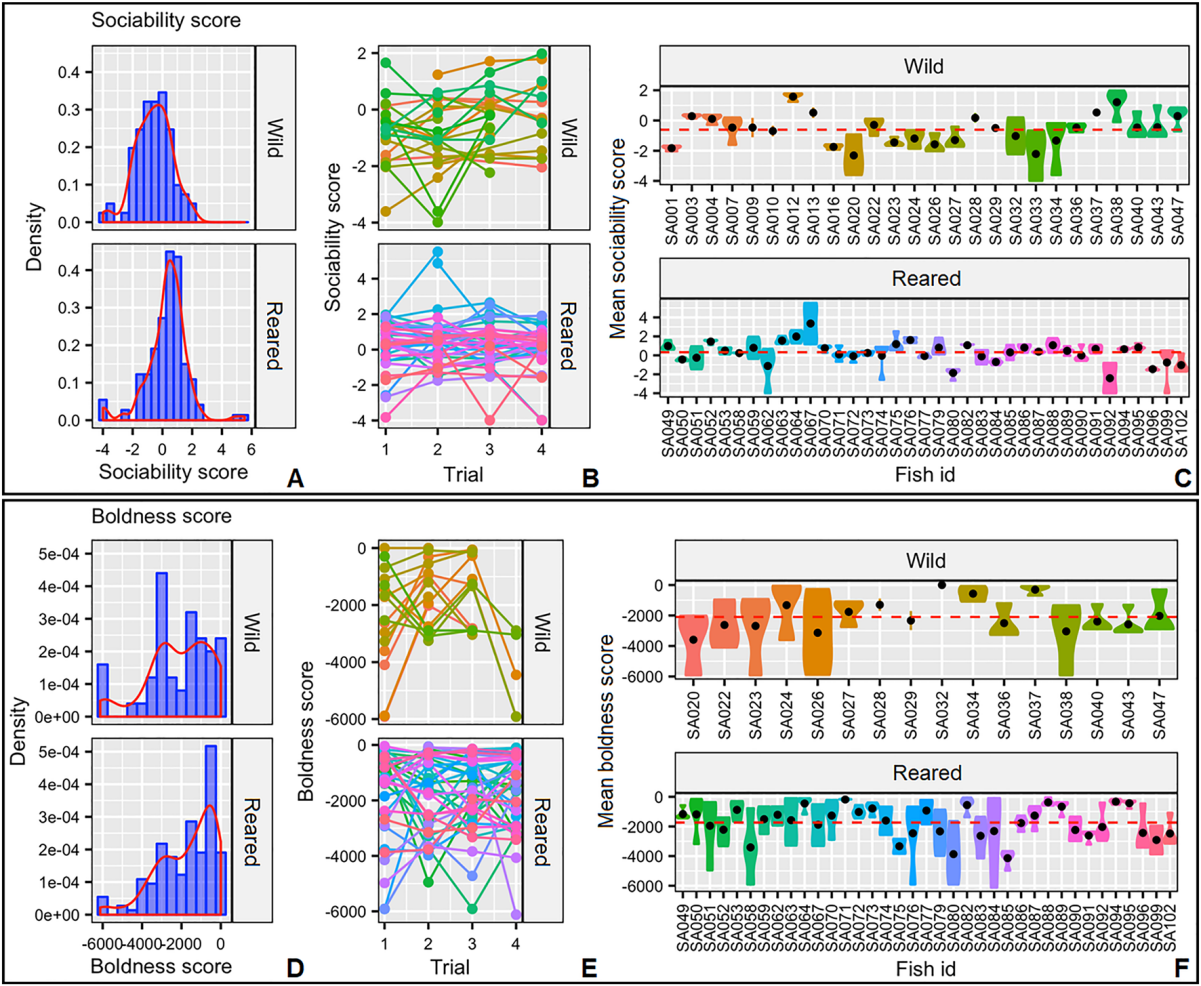
(**a**) Density plots of the sociability score, (**b**) individual’s sociability score per trial and (**c**) individual’s mean sociability score (black dot) for the two samples of gilthead seabream (*Sparus aurata*). Red dashed line represents the respective sample average; (**d**) Density plots of the boldness score, (**e**) individual’s boldness score per trial and (**f**) individual’s mean boldness score (black dot) for the two samples of gilthead seabream. Red dashed line represents the respective sample average.

Adjusted*-R* estimate for sociability in wild and reared samples was significant and averaged 0.53 with a BIC ranging from 0.49 to 0.60 (Table 2), confirming consistent repeatability for social behavior between individuals and across time (Fig. 3a–c). Individual behavioral variation ranged from the most social individual (SA067; Fig. 3c) that interacted for 35 min (of the one-hour test) with the other individual to the most asocial one (SA092; Fig. 3c) that did not show any interaction. The analysis of the potential covariates affecting sociability did not show any significant differences due to the experimental trial or individual length [LMM; *p* = 0.728 (trial); *p* = 0.522 (length); Table 2]. The LMM showed significant differences between the samples (*p* < 0.001), with the reared samples showing significantly higher social behavior with conspecifics (Fig. 3).

The adjusted*-R* estimate for boldness was the only one below the reference average R-score of 0.37^9^, resulting 0.25. However, it was significant, with a BIC ranging from 0.15 to 0.34 (Table 2), highlighting a repeatable boldness behavior in juvenile gilthead seabreams that is, most of the time, consistent between individuals and across time (Fig. 3d–f). Individuals varied greatly in boldness, where the boldest individual (SA037; Fig. 3f) presented the lowest latency time to feed (64 s), while the shyest individual (SA085; Fig. 3f) displayed a higher latency time to feed (more than 1 h). No covariates affected individual boldness [LMM; *p* = 0.698 (trial); *p* = 0.958 (length); *p* = 0.374 (trial); Table 2].

**Figure 4:**
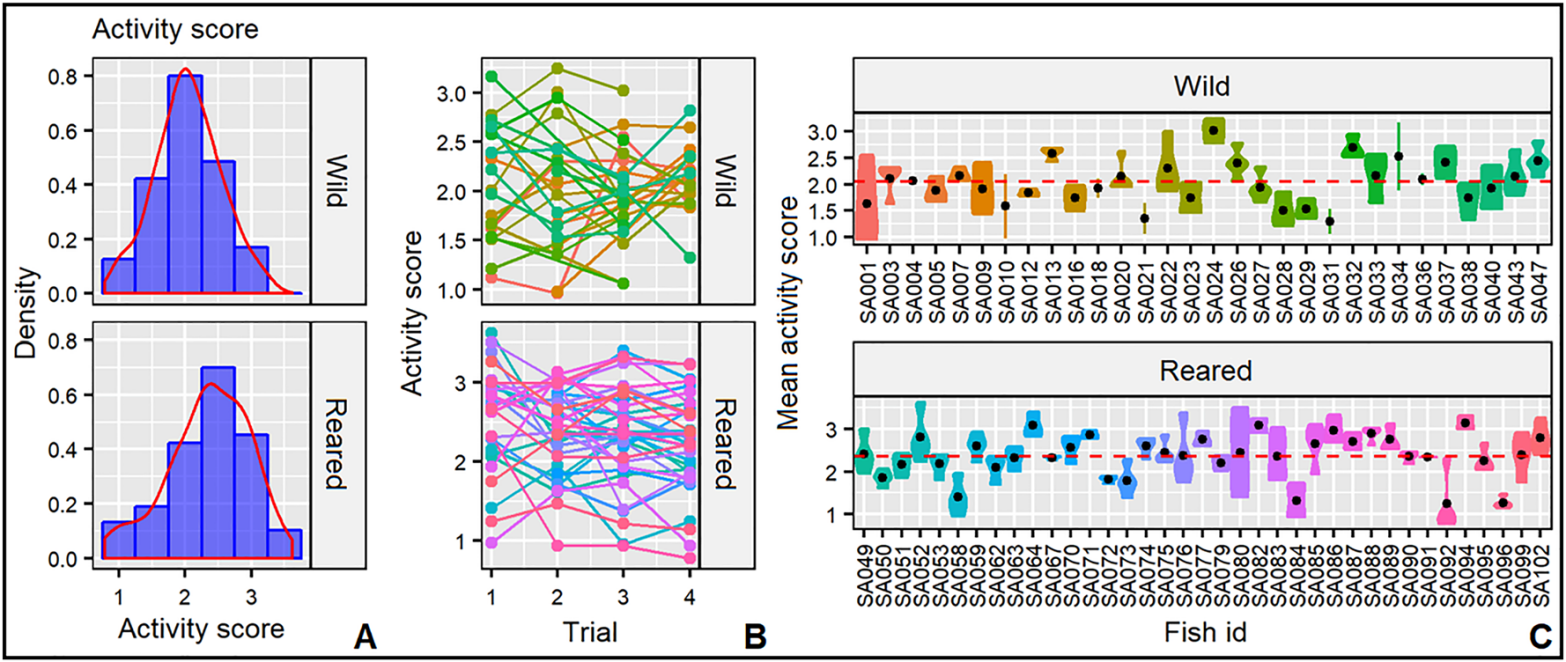
(**a**) Density plots of the activity score, (**b**) individual’s activity score per trial and (**c**) individual’s mean activity score (black dot) for the two samples of gilthead seabream (*Sparus aurata*). Red dashed line represents the respective sample average.

Finally, for activity, adjusted-R estimate in wild and reared samples averaged 0.54 with a BIC ranging from 0.47 to 0.58 (Table 2), indicating repeatability in activity behavior in juvenile gilthead seabreams. The most active individual (SA024; Fig. 4c) showed a maximum traveled distance of 7.7·10^6^ pixels (4.6·10^7^ cm), while the less active individual (SA096; Fig. 4c) only traveled 3.1·10^4^ pixels. The analysis of the potential covariates affecting activity showed no effect of individual’s length (LMM; *p* = 0.434; Table 2). The LMM indicated significant differences in activity levels of gilthead seabreams depending on the trial (*p* < 0.05; Table 2), with a relative decrease in activity with the trial progress. Also, significant differences were observed between samples (LMM; *p* < 0.01; Table 2), where reared individuals showed significantly higher swimming activity (Fig. 4).

### Behavioral syndromes

The phenotypic correlation decomposition through bivariate LMMs detected positive among-individual correlations between exploration-sociability (*rind* = 0.58 [0.28, 0.80]; Table 3) and exploration-activity (*rind* = 0.65 [0.37, 0.80]; Table 3) paired traits, suggesting the existence of two behavioral syndromes in juvenile gilthead seabreams. Accordingly, exploratory individuals with a tendency to investigate novelty also displayed a more social and active behavior, interacting more with conspecifics and showing a higher frequency of movement (Table 3).

**Table 3:** Between (*r*_*ind*_), within individual (*r*_e_) and phenotypic (*r*_p_) correlations (plus Bayesian credibility interval, BCI) between paired behavioral traits from the five personality axes examined: exploration (score), aggressiveness (approaches), boldness (score), sociability (score) and activity (score); using bivariate LMMs (five models).

Traits	Exploration	Aggressiveness	Boldness	Sociability	Activity
Between-individual correlations (*r*_*ind*_)					
Exploration	–	–	–	–	–
Aggressiveness	0.16 [− 0.20, 0.48]	–	–	–	–
Boldness	0.58 [− 0.58, 0.93]	− 0.02 [− 0.57, 0.62]	–	–	–
Sociability	**0.58 [0.28, 0.80]**	0.26 [− 0.11, 0.57]	0.34 [− 0.12, 0.80]	–	–
Activity	**0.65 [0.37, 0.80]**	− 0.08 [− 0.40, 0.25]	0.06 [− 0.33, 0.40]	0.25 [− 0.02, 0.47]	–
Within-individual correlations (*r*_*e*_)					
Exploration	–	–	–	–	–
Aggressiveness	− 0.17 [− 0.32, − 0.01]	–	–	–	–
Boldness	0.15 [− 0.01, 0.34]	− 0.10 [− 0.23, 0.04]	–	–	–
Sociability	−0.01 [ −0.13, 0.17]	− 0.07 [− 0.20, 0.10]	0.07 [− 0.10, 0.24]	–	–
Activity	**0.22 [0.05, 0.36]**	0.01 [–0.14, 0.17]	0.03 [− 0.13, 0.22]	− 0.10 [− 0.26, 0.05]	–
Phenotypic correlations (*r*_*p*_)					
Exploration	–	–	–	–	–
Aggressiveness	0.01 [− 0.25, 0.27]	–	–	–	–
Boldness	0.12 [− 0.01, 0.54]	− 0.10 [− 0.23, 0.04]	–	–	–
Sociability	**0.26 [0.02, 0.50]**	0.08 [− 0.16, 0.34]	0.06 [− 0.07, 0.47]	–	–
Activity	**0.42 [0.18, 0.58]**	− 0.03 [− 0.24, 0.21]	0.01 [− 0.08, 0.12]	0.11 [− 0.13, 0.32]	–

Correlations in bold were assumed to be significant (DIC between constrained and unconstrained model > 2).

## Discussion

In this study, we detected the existence of behavioral types (BTs) and syndromes, and found consistent differences in behavior between two different samples (wild and reared) of gilthead seabream under laboratory conditions. Adjusted-*R* estimates for all five behavioral traits were significant and showed consistent between-individual differences in juvenile gilthead seabreams. Exploration and activity were also significantly affected by the experimental trial, respectively increasing and decreasing along the four trials. We did not find a correlation between fish length and the studied behavioral traits. While the behavior of reared gilthead seabreams has been largely studied due to the interest for this species in the aquaculture industry, this is the first attempt to describe behavioral patterns under controlled conditions for wild individuals, providing novel insight into the behavioral effects of domestication on this species. The examination of the covariates affecting behavioral variation for the different axes in gilthead seabreams detected a significant effect associated with the sample (reared or wild). We found that the variability in BTs related to aggressiveness is drastically different between wild and reared individuals, with more aggressive individuals observed in the reared sample. Moreover, the samples differed in their levels of sociability and activity, the reared individuals being more social and active than the wild ones. The phenotypic correlation decomposition by paired behavioral traits detected significant between-individual correlations among exploration-sociability and exploration-activity, identifying two behavioral syndromes in juvenile gilthead seabreams. Our work provides the first attempt to assess the repeatability of behavior in wild and reared gilthead seabreams, providing interesting insights into the biology of this important species with implications for fisheries and aquaculture.

Besides having obtained relevant results about gilthead seabream behavior, this study proved the efficiency and adaptability of our fish tracking system^30^. The use of deep learning permitted us to carry out experiments in enriched (with the shelter and the sandy bottom) and changing (when introducing a novel object) behavioral arenas without having to design manually traditional computer vision algorithms that require problem-specific feature selection and extraction^49^. By simply incorporating more representative images (of another fish species or arena’s set-up) into the training process, our neural network can easily learn and generalize to new contexts, making it a versatile tool for a wide range of research applications. We, therefore, provide a non-invasive and highly adaptable tool to automatically quantify behavioral data from experimental arenas.

For all the five behavioral traits, we found consistent differences among-individuals. We observed that the exploration repeatability in juvenile gilthead seabreams (0.46 [0.37, 0.50]) fits well inside the range of adjusted-R estimates from other fish species like the convict cichlid (*Amatitlania siquia*)^50^ and the zebrafish (*Danio rerio*)^51^. Exploratory behavior plays a critical role in animal ecology, as demonstrated in the case of the argentine ant (*Linepithema humile*), where individual differences in this behavior are linked to important ecological processes such as gene expression, colony nest selection, and resource exploitation strategies^52^. In fish ecology, it can play a significant role in situations where individuals escape from fish farms into natural environments. As these individuals disperse, their exploratory behavior may influence the likelihood of genetic and ecological consequences, including the spread of diseases^53^. Contrasting our results, previous research on gilthead seabream^25^ did not show consistency in individual exploration-avoidance response via a novel object test. In this study, as stated by the authors themselves, the observed inconsistency in exploratory behavior may be partially attributed to the small size of the behavioral arenas utilized that may have hindered the observation of exploratory behavior. Moreover, the absence of a refuge in the behavioral arenas could make it impossible for a fish to hide, thus making it more difficult to detect any potential low exploratory behavior. Also, for aggressiveness, the repeatability (0.41 [0.31, 0.49]) approximates to the adjusted-R estimates from other fish species such as the green swordtail (*Xiphophorus helleri*)^42^, the bluefin killifish (*Lucania goodei*)^54^ and the zebrafish^55^. It is probable that aggressiveness in gilthead seabream*,* like in other animal species, plays an important role in modulating their wild populations, establishing hierarchical differences and the distribution of resources between individuals^4, 37, 54^. Sociability is also closely linked to the structure of a population and the competitive abilities of its individuals, where asocial individuals and poor competitors tend to disperse^38^. The repeatability score of sociability in gilthead seabreams (0.53 [0.49, 0.60]) was similar to other fish species like the three-spined stickleback^56^, and the guppy (*Poecilia reticulata*)^57^. Boldness showed the lowest repeatability scores in our tests (0.25 [0.15, 0.34]), even if the consistency of boldness-related traits has also been described in juvenile reared gilthead seabreams^25^ and in other species^58–60^. Finally, the observed activity repeatability in juvenile gilthead seabream (0.54 [0.47, 0.58]) displays the most repeatable behavior of our study and approximates the adjusted-*R* estimates of other fish species such as the pearly razorfish (*Xyrichtys novacula*)^48^.

Throughout the experimental trial, changes were observed in the exploratory and activity behavioral traits, with exploration increasing and activity decreasing. These patterns of changes in exploratory behavior have been observed in various species and may be associated with habituation effects. For instance, Sneddon, Braithwaite, and Gentle (2003) demonstrated that familiarizing animals with new objects could lessen their fear and promote exploratory behavior. In contrast, reduced exploration behavior may be a result of high cognitive abilities that allow animals to efficiently interact with their environment and acquire quickly effective strategies for navigation and exploitation^61^. For example, Adriaenssens and Johnsson^62^ found that six trials were enough for brown trout (*Salmo trutta*) to reduce significantly the search time for a cryptic prey by improving their ability to detect it on a matching background. The decrease in activity scores observed in successive trials of gilthead seabreams may also be attributed to habituation effects, as demonstrated in studies on Drosophila (*Drosophila melanogaster*)^63^ and the guppy^64^. These results underscore the significance of including the experimental trial factor as a potential confounding variable in the quantification of behavior.

In addition to the experimental trial, two other covariates were evaluated for correlation with behavior: the body length and weight of the individuals. These traits showed no relationship with the behavioral variation between individuals of gilthead seabreams in all the examined axes. This contrasts with other findings in the literature that reported positive correlations between body length and traits such as boldness and aggressiveness^42, 65, 66^. However, our results are consistent with other studies on different fish species, where individual differences in aggressiveness, boldness and sociability surpassed the body length effect^67, 68^. We attribute the lack of differences in behavior between different body lengths (and weights) to the limited range [10.1–14.5 cm] of sizes considered in this study, as we focused only on juveniles. Therefore, we recommend extending our work to larger (and adult) individuals to fully discard a relationship between personality traits and body length in this species.

We found consistent individual differences between wild and reared gilthead seabreams in aggressiveness, sociability and activity, with reared individuals displaying lower variance in aggressiveness and higher levels of sociability and activity. We, therefore, provide evidence of a plausible domestication effect that favors “proactive” individuals (in opposition to “reactive”), defined as more aggressive, social, active, bold, and explorative^4, 51^. The animal farming industry exerts a type of selective pressure that is very different from that of other domestication processes. Unlike companion animals, farmed animals continue to compete for limited resources like food and space^69^. In this context and given that the industry selects for large and fast-growing individuals, a "proactive" behavioral profile may be favored in the selection process^70^. This could be particularly evident in the aquaculture industry, where fish have limited contact with humans, making other behavioral aspects such as empathy and tameness less relevant. The results of aggressiveness require detailed attention. While the results of the LMM fitted to the aggressiveness scores suggest slightly higher levels of aggressiveness in reared fish (consistent with other studies in other species^71, 72^), the variance showed on this trait by the two samples was dramatically different limiting the power and robustness of this result. In fact, the distribution of the aggressiveness scores in the reared sample is much narrower than in the wild one, with the absence of both tame and highly aggressive individuals in the reared sample. The aquaculture industry could have produced a stabilizing selection that favors the average or middle levels of aggressiveness in reared fish. Alternatively, the reared sample considered in this study could come from the same genetic strain and, thus, could have similar genetic-induced behavior. However, this pattern was not consistent with the other four traits measured. Further corroboration is needed by exploring different well-known genetic strains in this species. Enhanced levels of sociability and activity caused by domestication have also been observed in juvenile zebrafish, where the reared sample was more social and showed a higher level of activity than the wild one^73^. Also, for these traits, and especially for activity, further studies are needed to control for the stress that may alter the behavior of a wild population placed into captivity. Understanding the effects of domestication on behavior may be a crucial step to improve fish welfare in the aquaculture industry (an aspect that strongly influences the production and the commercialization of reared fish), to prevent environmental disasters (adults or eggs escapes could alter the fitness of wild populations) and to understand the ecological consequences of BTs in wild animals.

Lastly, we have found that some of the BTs observed in our species covary resulting in behavioral syndromes. Both BTs and syndromes have major implications for the ecology and evolution of populations by constraining the ability of animals to behave optimally in all situations^1^. Here, we have detected two behavioral syndromes in juvenile gilthead seabreams in wild and reared samples along the axes of exploration-sociability and exploration-activity. Therefore, animals that exhibit a greater inclination towards exploring novel stimuli tend to be more socially and physically active, as evidenced by observations across multiple species^74, 75^. Previous research by Sánchez–Muros et al*.*^27^ found connections between the exploratory behavior and activity levels of reared gilthead seabreams, despite the lack of the statistical significance in correlations between behavioral traits. Moreover, similar to our results, exploratory behavior was related to sociability since they found high exploratory behavior in high stocking density tests. The role of BTs and syndromes in fisheries management is strictly connected to the resilience of a population to environmental changes^76^, especially when these are human-induced like exploitation by fishing pressure^11^. As common fisheries target in coastal areas, wild gilthead seabream populations are subject to fishing pressure that may lead to the elimination of extreme “proactive” types (i.e., more aggressive, social, and active), reducing the behavioral variability of the population^77^.

## Conclusions

To conclude, we hope that establishing a behavioral baseline for the gilthead seabream can help us understand how aquaculture and fisheries selection can affect its behavioral variability and challenge the survival of wild fish populations. We quantified the behavior of wild and reared gilthead seabreams along the five traits of fish behavior by performing standardized behavioral tests under laboratory conditions. We found consistent behavioral types at the individual level and consistent differences between two different samples, reared individuals being more similar in their aggressiveness scores, more social and more active than wild individuals. We also found that exploration-sociability and exploration-activity correlate, forming two behavioral syndromes. To fully comprehend the extent of our results, more investigation is needed to explore the behavior of adult gilthead seabreams and different well-known genetic strains. Understanding the behavior of wild and reared individuals of this species is crucial to improve our fisheries management programs, enhance fish welfare in fish farms and to control side effects in case of escape incidents.

## Data Availability

The datasets generated and analyzed during the current study are available in the DIGITAL.CSIC repository, https://doi.org/10.20350/digitalCSIC/14812.
